# DOES HAND GRIP STRENGTH CORRELATE WITH SHOULDER DYSFUNCTION AMONG OLDER WOMEN?

**DOI:** 10.1093/geroni/igad104.2255

**Published:** 2023-12-21

**Authors:** Ranyah Almardawi, Derik Davis, Denise Orwig

**Affiliations:** University of Maryland Baltimore/School of Medicine, Baltimore, Maryland, United States; University of Maryland School of Maryland, Baltimore, Maryland, United States; University of Maryland School of Medicine, Baltimore, Maryland, United States

## Abstract

**Background:**

Shoulder dysfunction and pain are common problems among older adults. Nearly 20% of those aged 65 and older reported shoulder pain in the 2011 National Health and Aging Trends Study. Shoulder pain and dysfunction may lead to the inability to carry out essential household and daily activities, burdening both patients and society. HGS is commonly tested and considered a reliable measure to assess overall muscle strength and function. Objective: The aim of this cross-sectional study is to determine the association between hand grip strength (HGS) and shoulder dysfunction. We hypothesize that HGS is correlated with shoulder dysfunction among older women.

**Methods:**

A total of 36 older women volunteers with an age range of 62 to 84 years were included. We assessed the HGS for dominant hands using Jamar Plus Digital Hand Dynamometer. We measured both hands twice and used the strongest measure for the dominant hand. Degree of shoulder dysfunction was self-reported using the American Shoulder and Elbow Surgeon survey (ASES) [0, worst; 100, best].

**Results:**

Participants had a mean age of 70.9 years (SD:5.7), mean grip strength of 18.2kg (SD:5.9), and mean ASES score of 86.8(SD: 17.2). A significant and positive correlation was found between HGS and ASES score (r=0.612, P=0.001).

**Conclusion:**

The moderate correlation of HSG with ASES score supports the hypothesis. The results suggest that future research is warranted to test the feasibility of HSG as a clinical screening tooling for shoulder dysfunction in larger diverse samples of older adults, including men and women.

